# Liver Transplantation for Acute Intermittent Porphyria is Complicated by a High Rate of Hepatic Artery Thrombosis

**DOI:** 10.1002/lt.22345

**Published:** 2012-01-25

**Authors:** Joanna K Dowman, Bridget K Gunson, Darius F Mirza, Simon R Bramhall, Mike N Badminton, Philip N Newsome, on behalf of the UK Liver Selection and Allocation Working Party

**Affiliations:** 1 Liver Unit, Queen Elizabeth Hospital BirminghamBirmingham, United Kingdom; 2 NIHR Biomedical Research Unit and Centre for Liver Research, University of BirminghamBirmingham, United Kingdom; 3 Department of Infection, Immunity, and Biochemistry, School of Medicine, Cardiff UniversityCardiff, United Kingdom

## Abstract

Acute intermittent porphyria (AIP) is an autosomal-dominant condition resulting from a partial deficiency of the ubiquitously expressed enzyme porphobilinogen deaminase. Although its clinical expression is highly variable, a minority of patients suffer recurrent life-threatening neurovisceral attacks despite optimal medical therapy. Because the liver is the major source of excess precursor production, liver transplantation (LT) represents a potentially effective treatment for severely affected patients. Using data from the UK Transplant Registry, we analyzed all transplants performed for AIP in the United Kingdom and Ireland. Between 2002 and 2010, 10 patients underwent LT for AIP. In all cases, the indication for transplantation was recurrent, biochemically proven, medically nonresponsive acute attacks of porphyria resulting in significantly impaired quality of life. Five patients had developed significant neurological morbidities such as paraplegia before transplantation. The median follow-up time was 23.4 months, and there were 2 deaths from multiorgan failure at 98 days and 26 months. Eight recipients were alive for 3.2 to 109 months after transplantation. Complete biochemical and symptomatic resolution was observed in all patients after transplantation. However, there was a high rate of hepatic artery thrombosis (HAT; 4/10), with 1 patient requiring regrafting. The effects of previous neuronal damage such as joint contractures were not improved by transplantation. Thus, impaired quality of life in the surviving patients was usually a result of preoperative complications. Refractory AIP is an excellent indication for LT, and long-term outcomes for carefully selected patients are good. There is, however, an increased incidence of HAT in these patients, and we recommend routine antiplatelet therapy after transplantation. Liver Transpl 18:195–200, 2012. © 2011 AASLD.

The porphyrias represent a heterogeneous group of mainly inherited disorders resulting from deficiencies of specific enzymes in the heme biosynthesis pathway.^1^, ^2^ Acute intermittent porphyria (AIP) is the most common of the 3 autosomal-dominant acute porphyrias and results from a partial deficiency of the ubiquitously expressed enzyme porphobilinogen (PBG) deaminase. Acute attacks are accompanied by the excess production of hepatic porphyrin precursors [PBG and 5-aminolevulinic acid (ALA)], which can be suppressed by the administration of intravenous hemin.

Epidemiological information collected in the United Kingdom as part of a project funded by the European Union Commission Public Health Executive Agency (European Porphyria Network) indicates that on average, 10 new AIP patients (including 8 women) present clinically each year. Approximately 10% of the female patients (and fewer of the male patients) may suffer recurrent acute attacks, which can include life-threatening neurovisceral attacks due to neuronal damage. The exact pathogenesis of the transient neuronal damage has not been established. The proposed mechanisms include ALA toxicity, a relative neuronal heme deficiency, neurotoxic products of ALA, PBG, or both, and a depletion of essential cofactors.^3^

Because the liver is the major source of excess precursor production,^3^ liver transplantation (LT) represents a potentially effective treatment. Only a small number of cases of LT for AIP have been reported so far.^4^-^6^ Using data from the UK Transplant Registry (which is maintained by National Health Service Blood and Transplant on behalf of transplant services in the United Kingdom and Ireland), we describe a larger national series with longer follow-up (median follow-up time = 23.4 months), and we include the indications and outcomes for all 10 LT procedures performed for AIP in the United Kingdom and Ireland since the first transplant in 2002.

## PATIENTS AND METHODS

This is a retrospective study of all known patients in the United Kingdom and Ireland who underwent transplantation for AIP; they were identified by a search of the UK Transplant Registry. The study was approved by the UK Liver Advisory Group. All patients received a definitive diagnosis of AIP that was based on clinical features and standard biochemical measurements, such as increased urinary PBG: creatinine ratios and normal fecal porphyrin excretion^7^; thus, other acute porphyrias such as hereditary coproporphyria and variegate porphyria were excluded.

The collected data included each patient's demographics, preoperative laboratory results, clinical status, date of transplantation, long-term survival, cause of death (when it was relevant), and postoperative complications. When it was possible, missing data were obtained by a review of the case notes or by communication with the appropriate liver unit. Patient survival was defined as the time between the initial transplant and death. Transplants were performed at 4 different centers within the United Kingdom and Ireland, although 6 of the 10 transplants (60%) took place at a single center (Birmingham).

## RESULTS

Between 2002 and December 2010, 10 patients underwent LT for AIP in the United Kingdom and Ireland. Only 5 of these patients were previously reported in the literature, and the follow-up was shorter.^4^-^6^ Nine patients (90%) were female, and this reflects the increased prevalence of symptomatic acute porphyria in women. The median age at transplantation (31 years, range = 18-50 years) was much lower than the median age of the traditional LT population.^8^

### Indication for Transplantation

Conventional scoring systems for assessing priority for LT are not applicable to patients with AIP, for whom parenchymal liver disease is unusual. Thus, the indication for LT in patients with AIP is recurrent, biochemically proven, medically nonresponsive acute attacks of porphyria resulting in significantly impaired quality of life. All the patients who underwent transplantation suffered recurrent acute attacks that manifested as abdominal pain, which was often associated with sympathetic overactivity (tachycardia and blood pressure lability), and peripheral neuropathies, which severely impaired their quality of life. Five patients suffered severe neurological symptoms that manifested as quadriplegia with respiratory failure, and they required admission to the intensive care unit for invasive ventilation. These symptoms were critical factors in the decision to perform transplantation for these individuals; however, after the early postoperative death of 1 patient who was quadriplegic and ventilator-dependent at the time of transplantation, subsequent patients were required to demonstrate independence from invasive ventilation before surgery. In most patients, acute attacks occurred at least monthly despite the regular (usually weekly) prophylactic administration of intravenous hemin. LT was proscribed for 1 patient (who was not a member of this series) because of a lack of venous access, which is a common complication in patients with AIP because of the requirement for regular intravenous hemin infusions; this patient subsequently died. In all cases, active porphyria was confirmed by an elevated urinary PBG:creatinine ratio in association with acute attacks.

### Posttransplant Survival and Outcomes

**Figure 1:**
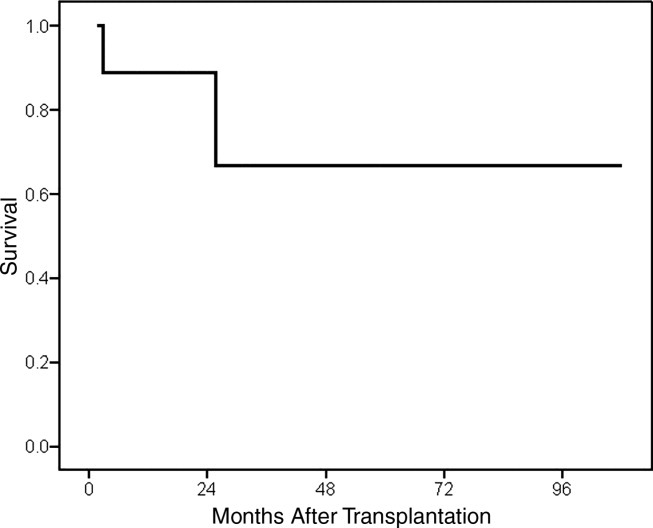
Survival after LT for AIP. Two of the 10 patients who underwent LT for AIP died (1 patient at 98 days and 1 patient at 26 months). The median follow-up time was 23.4 months.

The median follow-up time was 23.4 months (range = 3.2-109 months), and there were 2 recorded deaths at 98 days and 26 months. The patient who died at 98 days had been ventilator-dependent for several months before transplantation. This patient required the evacuation of a hematoma 2 weeks after transplantation, and renal failure, pressure sores, and recurrent chest and line sepsis developed; the patient never regained independence from intensive organ support and died from multiorgan failure. The death at 26 months was also a result of multiorgan failure, but no further details could be obtained. One patient underwent regrafting after 13 days for hepatic artery thrombosis (HAT) but remained alive and well 53 months later. The remaining 8 patients were alive for 3.2 to 109 months after transplantation (Fig. 1). The rates of survival for these patients are comparable to the 3-month and 5-year survival rates for elective adult LT at the reporting centers over the same time period (93% and 77%, respectively).

**Figure 2:**
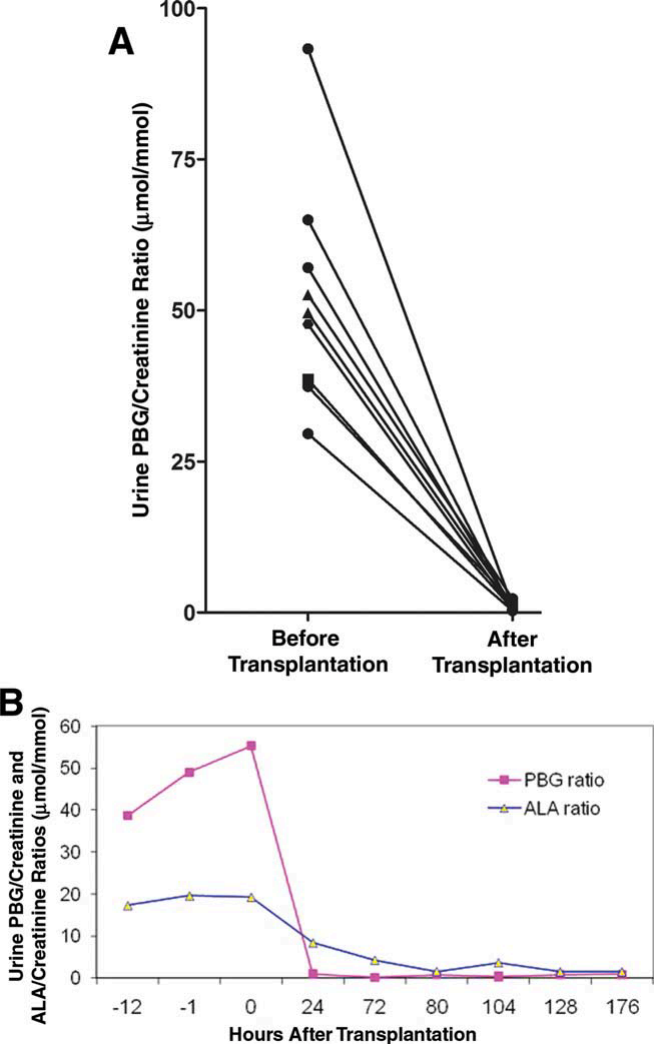
AIP activity after LT. (A) Urine PBG/creatinine ratios for 9 of the 10 AIP patients before and after LT. All 9 patients experienced a complete biochemical resolution of their disease (reference level < 1.5 μmol/mmol). The 10th patient had an elevated pretransplant ratio of 74.3 μmol/mmol, but no posttransplant level was available. However, this patient was asymptomatic after transplantation, and the posttransplant level was, therefore, expected to be normal. (B) Short-term urinary PBG/creatinine and ALA/creatinine ratios for a single AIP patient before and after LT. The urinary PBG level returned to normal within 24 hours and remained normal thereafter. The urinary ALA excretion level returned to normal within approximately 72 hours.

LT resulted in no further porphyria attacks for the recipients, who experienced a complete biochemical resolution of their urinary PBG excretion (Fig. 2A). Urinary PBG and ALA levels can be expected to return to normal within 24 and 72 hours of transplantation, respectively (Fig. 2B), and to remain normal thereafter. Monitoring PBG levels after transplantation is, therefore, unnecessary. One patient who underwent transplantation in 2002 at the center in Birmingham returned to full-time employment and gave birth to a healthy child.

**Figure 3:**
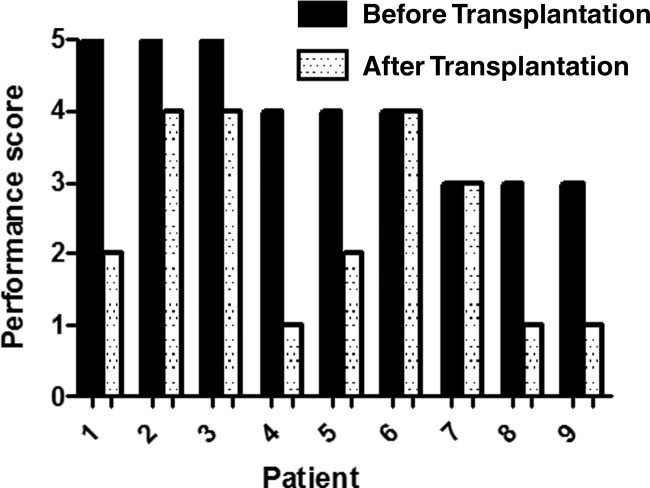
Pretransplant and posttransplant performance scores for 9 patients who underwent LT for AIP and had significant postoperative survival. The following scale is used to describe their quality of life: (1) the patient is able to carry out normal activities without restriction, (2) the patient is restricted only from physically strenuous activity, (3) the patient can move freely and is capable of self-care but is incapable of any form of work, (4) the patient is capable of only limited self-care and is mostly confined to a bed or a chair, and (5) the patient is completely reliant on nursing/medical care. All these patients were prevented from performing any type of work before LT; 6 patients were mostly confined to a bed or a chair or were completely reliant on medical/nursing care. LT led to significant improvements in the quality of life for most of the patients; their posttransplant limitations either depended on chronic neurological deficits sustained before transplantation or resulted from those deficits (eg, contractures).

There was no evidence of neurological deterioration in the perioperative period, which had been a theoretical concern. Although the performance scores improved after transplantation (see Fig. 3), there was no evidence of any marked improvements in longstanding neurological deficits or in complications due to those neurological deficits. These residual complications were most likely due to the effects of neuronal deficits (eg, contractures) rather than actual ongoing neuronal deficits, but nerve conduction studies would have been required for confirmation. The side effects of earlier interventions, such as tracheal damage from ventilation, may also remain.

Psychiatric manifestations such as depression are not uncommon in patients with AIP.^1^ Two of the 7 patients for whom these data were available were prescribed antidepressants before transplantation, and these drugs were continued postoperatively. Two other patients developed a benzodiazepine or opiate dependency, which required the implementation of a weaning protocol after transplantation. Although the patient with the benzodiazepine dependency was successfully treated, the patient with the opiate dependency, who suffered significant residual neurological damage, still had significant opiate requirements 6 months after transplantation. No psychiatric issues were identified in the other 3 recipients.

### Complications

Two patients required further surgery for early hemorrhaging, and 1 patient experienced a biliary leak at 36 days. HAT occurred in 4 patients. In 1 patient, this necessitated retransplantation 13 days after the original procedure; in the other 3 patients, HAT occurred 1, 8, or 9 months after transplantation. HAT was detected in the first patient on day 3 by an ultrasound scan performed for impaired liver function; the scan failed to show a donor hepatic artery. This was subsequently confirmed by an angiogram, which demonstrated an occlusion of the hepatic artery from just distal to the origin. This patient had received a right split liver graft; a small hepatic artery from the donor's superior mesenteric artery was noted perioperatively. Ultrasound and Doppler scans, which were repeated 1, 10, and 31 days after retransplantation, confirmed that the vessels remained patent. The second patient with HAT presented with fever, abnormal liver function, and abdominal pain 1 month after transplantation. A computed tomography (CT) scan demonstrated donor HAT with central ischemic changes in the liver. Subsequent ultrasound scans and a repeat CT scan confirmed these findings and revealed thrombosis of the hepatic artery 1 cm from its origin, some small collaterals at the porta hepatis, and bilomas in both liver lobes. At the time of this writing, the patient was awaiting retransplantation. The patient whose HAT was detected at 9 months also had a split liver graft and initially presented with jaundice. Initial imaging studies revealed a biliary stricture, and a subsequent CT scan suggested that this was secondary to HAT with collateralization. An angiogram demonstrated an occlusion of the common hepatic artery from its origin with several collaterals supplying the liver. Although this patient was initially relisted for retransplantation, subsequent clinical improvement and the normalization of liver function tests led to the patient's removal from the waiting list. Similarly, the patient who developed HAT at 8 months also developed good collateral circulation associated with normal liver function and thus did not require retransplantation. The presence of collaterals at the time of the HAT diagnosis in these 2 patients suggests that this complication may have occurred at an earlier time point but was undetected. Routine ultrasound scans are not usually performed for patients after transplantation in the absence of symptoms or abnormal liver function. However, these findings suggest that early and late posttransplant ultrasound scans should be considered in this group of patients to exclude asymptomatic HAT. Notably, the patient whose HAT was detected at 9 months had been taking both aspirin and fixed-dose warfarin since transplantation because of the nature of the hepatic arterial anastomosis, although the international normalized ratio was not in the therapeutic range. The 2 patients with early HAT detection took neither of these agents. None of the 3 HAT patients for whom perioperative details were available received any intraoperative transfusions of blood, fresh frozen plasma, or platelets.

Progressive renal disease has been observed in patients with AIP, and this may be attributable to chronic hypertension, direct porphyrin precursor nephrotoxicity, or causes unrelated to porphyria.^9^, ^10^ In our cohort, 2 patients had preexisting renal impairment, and 1 of these patients required renal transplantation 39 months after LT. The progression to end-stage renal failure in this case was attributed to tacrolimus nephrotoxicity on clinical grounds. Another patient had temporary tacrolimus-induced nephrotoxicity within the first 6 months after transplantation, but the patient's renal function returned to normal after the immunosuppressive regimen was switched. The creatinine clearance rate for another patient had fallen to 75 mL/minute 18 months after transplantation. Renal failure also contributed to the multiorgan failure that led to the death of 1 patient at 98 days. Although data on posttransplant renal function were unavailable for 2 patients, the available data suggest that the additional insults of surgery and nephrotoxic immunosuppressive agents may increase the risk of renal dysfunction in an already vulnerable cohort.

### Explant Histology

A histological analysis of the liver explants revealed that 7 of the 10 patients (70%) had moderate or severe siderosis, although the hepatic iron levels were not formally measured. This was likely due to previous treatments with hemin because siderosis is not a reported feature of AIP in the absence of such therapy.^11^ Several other minor and nonspecific histological abnormalities were also observed that were not readily attributable to AIP. These included a mild portal chronic inflammatory infiltrate (n = 1), mild portal fibrosis and nonspecific inflammation (n = 2), and a mild ductular reaction (n = 1). Mild granulomatous inflammation with architectural changes and biliary features suggestive of a possible vascular etiology was also reported for another explant.

An increased risk of hepatocellular carcinoma^12^, ^13^ and cirrhosis^14^ has been previously reported for patients with AIP, although cirrhosis appears to be rare.^15^ Neither cirrhosis nor hepatocellular carcinoma was observed in our cohort.

## DISCUSSION

The data in this study confirm that LT for AIP patients is essentially a curative procedure: 8 of the 10 patients who underwent transplantation for AIP were alive and free from porphyria symptoms at the time of this writing. Although there are improvements in the performance status, there are no marked improvements in any long-term residual neurological deficits after transplantation.

Recurrent attacks of porphyria in patients with AIP cause major impairments in their quality of life and complications such as motor peripheral neuropathies, which can lead to respiratory and bulbar paralysis and death. The decision to consider LT is based on the frequency and severity of the attacks as well as the patient's age and quality of life. Because not all patients inheriting the genetic mutation for AIP develop the clinical syndrome, it is important to demonstrate the biochemical activity of AIP by raised urinary PBG:creatinine ratios before patients are considered for transplantation. Historically, it was believed that symptoms might improve after the age of 40 years, so it was suggested that there might be value in deferring the decision for patients approaching this age. However, on closer examination, this premise does not appear correct, and it needs to be weighed against the likelihood of ongoing life-threatening acute attacks, particularly when intravenous access for treatment with hemin is limited. Specifically, we strongly suggest a referral for LT assessment for any patient suffering severe progressive neurological symptoms as part of an acute attack despite adequate medical therapy. Our experience confirms that by preventing further attacks, LT allows the stabilization of neurological symptoms and often leads to improvements, and most patients have significantly improved quality of life after LT. A patient who was quadriplegic and ventilator-dependent because of a motor neuropathy at the time of LT did not survive, and this suggests that transplantation should be deferred for these patients until they are successfully weaned from the ventilator. Auxiliary LT has been suggested as a possible option for these patients; however, the volume of additional functioning liver tissue required to fully prevent the overproduction of porphyrin precursors by the native liver is unknown, and the value of this procedure in patients with AIP remains to be established.

The degree of vascular access is a potentially important consideration in the timing of LT for these patients. Indwelling central venous catheters, which are used to administer intravenous hematin for the treatment of acute attacks, frequently lead to vascular thromboses, and multiple vascular thromboses can complicate LT. LT was deemed to be too risky for 1 patient (who was not a member of this series) because of multiple major vein thromboses (including the superior vena cava, both internal jugular veins, and the left common femoral and left subclavian veins), and this individual subsequently died from sepsis after portacath insertion. We suggest that the vascular access should be evaluated as part of the LT assessment, and our practice requires that at least 2 of the 6 major veins, including 1 internal jugular vein, be patent for LT.

HAT occurred in 4 of the 10 patients (40%) who underwent transplantation for AIP. HAT was detected early in 2 patients (within the first 36 days) and later in the other patients (at 8 and 9 months). Although HAT is more common in the pediatric population,^16^ it has been reported to complicate 3% to 5% of adult orthotopic LT procedures.^16^-^18^ This is consistent with the data from the UK Transplant Registry, which has reported a 2.9% incidence of early HAT (occurring within 90 days of transplantation), and with the data from the center in Birmingham, which has reported a 3.1% incidence of late HAT (occurring after 90 days) during the period of study. The high incidence of HAT in AIP patients was previously unreported and is considerably higher than the rate expected for the general LT population. Although the underlying risk factor is unclear, all the patients had received multiple doses of hemin for the treatment of acute attacks immediately before LT. Technical factors may also have been involved; for example, a small donor hepatic artery was noted in 1 patient, and 2 patients received split liver grafts. The intraoperative use of blood products, including fresh frozen plasma and platelets, is frequent in LT. However, no such products were administered perioperatively to the 3 HAT patients for whom these data were available, so this is unlikely to have been relevant. We have amended our treatment recommendations to include routine antiplatelet therapy after transplantation and anticoagulation with warfarin for patients who develop HAT while they are receiving antiplatelet therapy.

Although renal dysfunction was infrequently observed in our cohort, it is common in patients with AIP,^9^, ^10^ and the successful combination of renal transplantation and LT has recently been reported for 2 AIP patients with coexisting chronic renal failure.^19^

In conclusion, although the decision to transplant is often difficult and requires the consideration of several factors, refractory AIP is an excellent indication for LT with good long-term outcomes for carefully selected patients. However, because of the increased incidence of HAT in this cohort, antiplatelet therapy after transplantation is recommended.

## Abbreviations

AIP: acute intermittent porphyria
ALA: 5-aminolevulinic acid
CT: computed tomography
HAT: hepatic artery thrombosis
LT: liver transplantation
PBG: porphobilinogen
